# Characterization of DTI Indices in the Cervical, Thoracic, and Lumbar Spinal Cord in Healthy Humans

**DOI:** 10.1155/2012/143705

**Published:** 2012-01-05

**Authors:** Rachael L. Bosma, Patrick W. Stroman

**Affiliations:** ^1^Centre for Neuroscience Studies, Queen's University, Kingston, ON, Canada K7L 3N6; ^2^Departments of Diagnostic Radiology and Physics, Queen's University, Kingston, ON, Canada

## Abstract

The aim of this study was to characterize *in vivo* measurements of diffusion along the length of the entire healthy spinal cord and to compare DTI indices, including fractional anisotropy (FA) and mean diffusivity (MD), between cord regions. The objective is to determine whether or not there are significant differences in DTI indices along the cord that must be considered for future applications of characterizing the effects of injury or disease. A cardiac gated, single-shot EPI sequence was used to acquire diffusion-weighted images of the cervical, thoracic, and lumbar regions of the spinal cord in nine neurologically intact subjects (19 to 22 years). For each cord section, FA versus MD values were plotted, and a k-means clustering method was applied to partition the data according to tissue properties. FA and MD values from both white matter (average FA = 0.69, average MD = 0.93 × 10^−3^ mm^2^/s) and grey matter (average FA = 0.44, average MD = 1.8 × 10^−3^ mm^2^/s) were relatively consistent along the length of the cord.

## 1. Introduction

Diffusion tensor imaging (DTI) allows for the *in vivo*  examination of the extent of damage to white matter microstructure which may enable the detection and diagnosis of subtle injuries and may provide a means of monitoring the effects of a therapeutic intervention. The applications of this technique for characterizing the structural changes that result from lesions in the brain have become well established [1]. More recently, DTI has also been applied to the spinal cord and has been demonstrated to be a similarly valuable tool for assessing the extent of white matter damage in numerous spinal cord-related conditions including multiple sclerosis [2, 3], amyotrophic lateral sclerosis [4, 5], myelitis [6, 7], and spinal cord injury (SCI) [8, 9]. However, despite its potential as a clinical tool, it is first necessary to establish reference values of fractional anisotropy (FA, which describe the degree to which a single diffusion orientation is dominant) and mean diffusivity (MD, which describes the overall diffusivity) in healthy populations in order to be able to properly interpret DT images acquired in patients. Furthermore, estimating the consistency of DTI indices across different regions in the healthy spinal cord is required for proper group comparisons between heterogeneous patient populations and healthy controls. The aim of the present study was therefore to characterize and compare DTI indices across the cervical, thoracic, and lumbar regions of the healthy spinal cord. 

Several studies have examined FA and MD values at different levels within the cervical spinal cord [10, 11]. Results from these studies suggest that there is, although subtle, variance in DTI indices within the cervical cord alone, highlighting the importance of examining whether there are more remarkable differences between the cervical, thoracic, and lumbar regions of the cord [12]. To our knowledge, only one other study has examined DTI indices from the whole cord, and comparisons of measures between different sections of the cord revealed a rostral to caudal decrease in FA values (0.95–0.2) while mean diffusivity remained constant along the cord. Additionally, measurements of FA from individual regions of interest (ROIs) were consistently greater in white matter (0.68) compared to grey matter (0.47) and CSF (0.15). However, this study did not employ cardiac gating, and the data were obtained with relatively low resolution (1.6 × 1.6 × 5 mm) and involved subjects with a wide age range, thereby limiting the conclusions that could be made. Nonetheless, this study revealed important characteristics of cord DTI indices that warrant further investigation. 

Application of this technique to the human spinal cord is technically challenging due to the small cross-section of the cord, pulsatile cord motion, and field inhomogeneities caused by susceptibility variations from nearby vertebrae. High axial-plane resolution is required to reduce partial volume effects in which the signal arises from both the cord and the cerebral spinal fluid (CSF) or from both white and grey matter, given that the spinal cord structure is, in effect, inverted from that of the brain, with white matter surrounding a central core of grey matter. Furthermore, cord motion, a major source of which is the cardiac induced pulsatile motion of the CSF fluid surrounding the cord [13], greatly influences measurements of water self-diffusion in the spinal cord [14, 15]. For example, FA values measured from the cervical cord using cardiac gating [16] differ from values reported from the same region of the cord without gating (0.83 versus 0.70, resp.) [17]. Therefore, the current study aims to address these methodological limitations by acquiring diffusion tensor images with a high spatial resolution (1.2 × 1.2 × 3 mm) and by implementing cardiac gating as a means of further reducing effects of spinal cord movement within the spinal canal, and of CSF flow-related artifacts. 

It is important to know whether DTI indices, such as the mean diffusivity (MD) and the fractional anisotropy (FA), are consistent across cervical, thoracic, and lumbar regions of the healthy cord. This knowledge will indicate whether changes in DTI indices as a result of trauma at any level can be characterized relative to values obtained in the same patient from distant regions of the cord that were presumably not affected by the trauma. Such a comparison is necessary unless normative data is available from well-matched control subjects with the exact same DTI acquisition parameters. Therefore, we compared DTI indices, including fractional anisotropy (FA) and mean diffusivity (MD), for three sections of the healthy spinal cord: cervical, thoracic, and lumbar.

## 2. Methods

### 2.1. Subjects

Participants included 9 adults (five males and four females) with no prior history of neuropathology. The age of the participants ranged from 19 to 22 years (mean age 19.1 years) in order to avoid age-related variations [10]. Consent was obtained from all participants, and all participation was voluntary. This study approved by the Health Research Ethics Board was in accordance with the Tri-Council Policy Statement on Ethical Conduct for Research Involving Humans.

### 2.2. Image Acquisition

**Figure 1:**
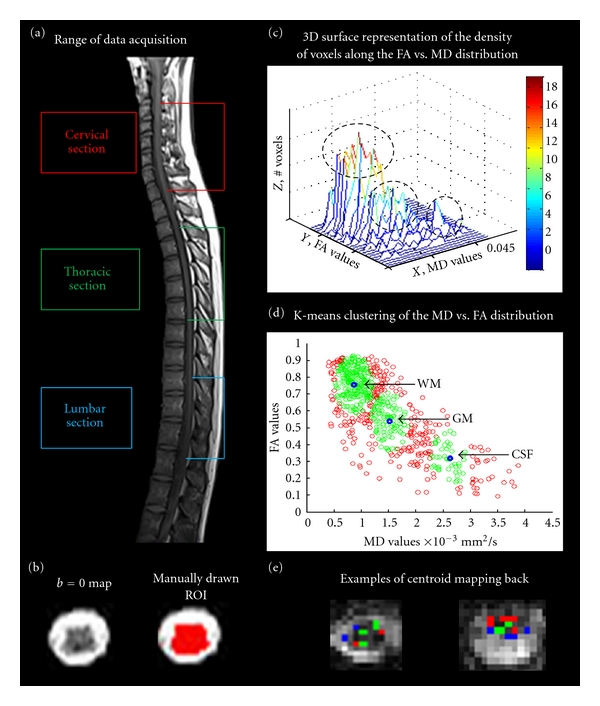
(a) Sagittal view of the spinal cord indicating the cervical, thoracic, and lumbar regions acquired in the diffusion-weighted data. (b) An example of a *b* = 0 map and the same *b* = 0 map with an ROI manually drawn on the image to indicate the boundaries of the spinal cord. (c) An example of a 3D-surface representation of the density of voxels along the FA versus MD distribution from the cervical cord of one subject. The height of the surface (*z*) reflects the number of voxels with overlapping MD (*x*) and FA (*y*) values on the plot. From this example, it is apparent that voxels cluster in three distinct groups, the highest density of voxels with high FA and low MD values, the middle cluster with low FA and low MD values, and a small third cluster with low FA and high MD values. (d) An example of the FA versus MD voxel distribution in the cord from one subject in the cervical region of the spinal cord. All voxels were plotted (red) and a k-means clustering method partitioned the data into three distinct clusters. The center of the cluster (or “centroid”) is shown in blue and is the mean FA and MD of each cluster. Clusters were further restricted to include only voxels which fell within a threshold distance from the centroid (green), thereby, excluding the voxels with the greatest amount of partial volume effects. Clusters with high FA values and low MD values are assigned to white matter (WM), clusters with low FA values and low MD values are assigned to grey matter (GM), and clusters with low FA values and very high MD values are assigned to cerebral spinal fluid (CSF) and noise. (e) Two examples of the spatial location of the centroid mapping back. The green voxels indicate where the grey matter cluster mapped back to, while the red indicates white matter voxels and the blue is CSF. Grey matter voxels (green) were located in the center regions of the cord and extended outwards towards the dorsal and ventral horns while white matter clusters (red) were located to areas outside of the grey matter regions. CSF clusters (blue) consistently mapped to the outer boundaries of the cord.

All imaging was performed with a 3T Siemens Tesla whole-body MRI (Magnetom Trio; Siemens, Erlangen, Germany). Radiofrequency excitation was performed with a body coil, while a spine phased-array coil, head coil, anterior and posterior neck coils, and a flexible body coil positioned over the chest were used as receivers, depending on the level of the cord being imaged. The imaging protocol consisted of a 3-plane fast gradient-recalled echo sequence to provide initial localizer images and then T_2_-weighted coronal and sagittal localizers were acquired with a half-fourier single-shot fast spin-echo (HASTE) sequence for more precise anatomical position references for determining the spinal cord regions. A single-shot spin echo EPI sequence was used to collect diffusion-weighted images of the cervical, thoracic, and lumbar regions of the spinal cord (Figure 1(a)). For each cord region, images were acquired in seven separate imaging series, each consisting of 4 slices, 3 mm thick, separated by an 18 mm gap. In each successive acquisition, the slice positions were shifted by 1 slice thickness, so that after all 7 acquisitions, a total of 28 contiguous slices were obtained. Only 4 slices were imaged at a time to accommodate the cardiac-gating method, as described below. The cervical section spanned the 2nd to 7th cervical vertebrae (C2–C7), the thoracic section spanned from the 3rd to 8th thoracic vertebrae (T3–T8) while the lumbar section of the cord spanned from the 10th thoracic to 1st lumbar vertebrae (T10-L1) (Figure 1(a)). Images were acquired with the following parameters: TE = 103 ms, TR determined by the cardiac gating, SENSE parallel imaging with an acceleration factor of 2, and a matrix size of 128 × 128. Diffusion weighting was applied in 20 directions with a *b*-value = 700 s/mm^2^ and in one scan with *b* = 0 and had an in-plane resolution of 1.2 mm × 1.2 mm, and a slice thickness of 3 mm. Whereas *b*-values of 1000 s/mm^2^ are commonly used for brain DTI the lower SNR in the spinal cord, and challenges presented by the inhomogeneous magnetic field environment within the cord warrant a reduction of *b*-value to 700 s/mm^2^ with little cost in sensitivity [10]. Furthermore, with a lower *b*-value, shorter TE values are typically possible providing further increase in the signal-to-noise ratio (SNR). The choice of 20 diffusion directions was based on previous studies as this provides both good accuracy for the estimation of the diffusion tensor and maintains a relatively short acquisition time [18, 19]. Cardiac gating was applied to reduce the impact of spinal cord motion that result from pulsating CSF [13, 15]. The cardiac trigger delay was set at 200 ms after peripheral systole so that image acquisition occurred within 660 ms (the set TR value) of the most quiescent part of the cardiac cycle [15]. A long effective TR (four heart beats) was used so that fluctuations in the heart rate did not create fluctuations in T_1_-weighting and thereby affect the MR signal. Each section of the cord took approximately 17 minutes to image, depending on the heart rate, and the total imaging session took 1 hour. Finally, a whole-cord high resolution T_2_-weighted image was acquired for anatomical comparisons. 

### 2.3. Image Analysis and Statistics

All analyses were completed using custom-made software, written in MatLab (The MathWorks Inc., Natick, MA, USA). The complete set of 84 slices (3 regions, 28 slices each) was placed in order of the rostral-caudal position along the cord. The diffusion-weighted data for each voxel was used to construct a 3 × 3 diffusion tensor, and eigenvectors and eigenvalues of the tensors were calculated to determine the principal directions of diffusion and their magnitudes (*λ*_1_, *λ*_2_, and *λ*_3_), respectively. Mean diffusivity and fractional anisotropy were calculated for each voxel as follows [20]:

(1) $$\begin{array}{c} \text{MD}=\hat{\lambda }=\frac{1}{3}\left(\lambda_{1}+\lambda_{2}+\lambda_{3}\right),\\ \text{FA}=\sqrt{\frac{3}{2}\frac{\left[{\left(\lambda_{1}-\hat{\lambda }\right)}^{2}+{\left(\lambda_{2}-\hat{\lambda }\right)}^{2}+{\left(\lambda_{3}-\hat{\lambda }\right)}^{2}\right]}{\left(\lambda_{1}^{2}+\lambda_{2}^{2}+\lambda_{3}^{2}\right)}.} \end{array}$$

For each section of the cord, region-of-interest (ROI) maps were manually drawn on each *b* = 0 transverse slice to indicate the entire cord crosssection (Figure 1(b)). FA versus MD values from each voxel were then plotted for each section, resulting in a continuum of values which is reflective of voxels containing grey matter, white matter, CSF, and those containing mixed proportions of these tissues. The density of voxels along the FA versus MD distribution was plotted with a 3D-surface representation, with the height of the surface indicating the numbers of voxels with overlapping values on the plot, in order to facilitate the identification of separate clusters. Visual inspection indicated the presence of three clusters although this was anticipated based on the anatomical structure of the spinal cord (Figure 1(c)). A k-means clustering method (“kmeans” function in MatLab) was applied to partition the data into three clusters, and to determine the mean FA and MD of each cluster (i.e., the “centroids”). The clusters were then restricted to include only voxels which fell within a threshold distance (20% of the distance to the nearest adjacent centroid), thereby, excluding the voxels that were expected to have the greatest amount of partial volume effects because they fall between the centroids (Figure 1(d)). However, this restriction still retained more than 50% of the voxels within each cluster, as originally assigned. Clusters with high FA values, low MD values, and containing the largest number of voxels are assigned to white matter (WM), while clusters with low FA values, low MD values, and the intermediate number of voxels are assigned to grey matter (GM) [12, 21]. Clusters with low FA values, very high MD values, and the least number of voxels (given the ROI mask applied initially) are assigned to cerebral spinal fluid (CSF) and noise. Voxels contributing to each cluster were then mapped back on to the *b* = 0 maps to test whether or not they originated from the correct anatomical locations based on the cluster assignments (Figure 1(e)). Comparisons of the mean MD and FA values for each cluster, between cervical, thoracic, and lumbar regions, were made using a 2-tailed, one sample *t*-test with unequal sample sizes and unequal variance.

## 3. Results

**Figure 2:**
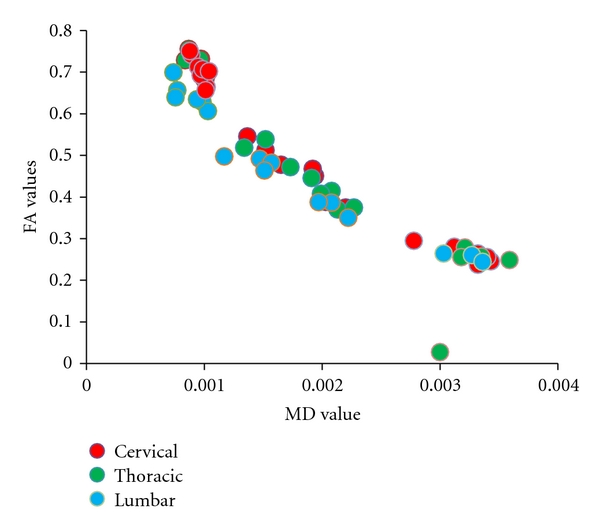
Centroid coordinates for each region of the cord, for each person. Centroid coordinates, which reflect the mean FA and MD for each cluster, remained relatively consistent along the length of the cord, specifically in the white matter cluster.

The 3D-surface representation of the density of voxels along the FA versus MD distribution reveals three peaks for the majority of subjects and in most regions of the cord: a large peak with high FA and low MD values, a smaller middle peak with low FA and low MD values, and a slight third peak (low FA and high MD) (Figure 1(c)). Visual inspection of the back mapping of voxels from white matter, grey matter, and CSF clusters indicates that these clusters are appropriately assigned and contain voxels from the respective tissues; however, in some cases, the correct assignment was unclear and appeared to be strongly affected by partial-volume effects (Figure 1(e)). The mean and standard deviations of the DTI indices across each region for each tissue type are summarized in Table 1. Clusters with high FA and low MD, which we have attributed to white matter, had relatively consistent FA and MD values along the cord (FA = 0.65–0.71, MD = 0.87–0.97 × 10^−3^ mm^2^/s). More specifically, differences in MD values for the cervical, thoracic, and lumbar cord were not statistically significant, while FA values differed only between the lumbar cord and the other cord regions. For grey matter clusters (with low FA and MD, FA = 0.44–0.45, MD = 1.7–1.9 × 10^−3^ mm^2^/s), no significant differences were evident in MD or FA values between the cord regions. Finally, the cluster attributed to CSF and noise cord (FA = 0.27–0.30, MD = 2.72–3.12 × 10^−3^ mm^2^/s) also demonstrated significant differences between MD values across all three sections of the cord. The FA versus MD distribution from each tissue type, for each person, demonstrates the consistency of the white matter indices between participants and along the cord (Figure 2). Comparatively, the grey matter indices show greater variation which suggests that this cluster may include several voxels that contain partial volume contamination between CSF and white matter. 

**Figure 3:**
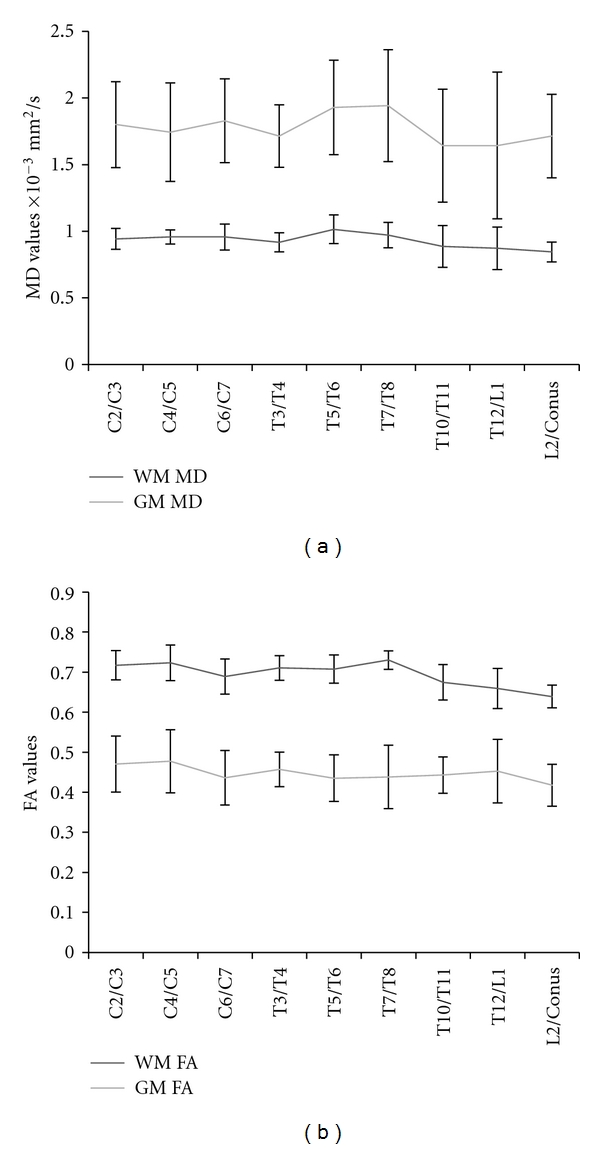
(a) MD values from the white matter and grey matter cluster averaged across subjects for each segment of the cord. (b) White matter and grey matter FA values averaged across subjects for each segment of the cord.

Diffusion properties in white matter and grey matter evaluated along the length of the entire spinal cord and are depicted in Figure 3. White matter FA values demonstrate a slight rostral to caudal decrease in value, while grey matter FA values are consistent along the cord. MD values remain relatively consistent along the length of the cord; however, there was a slight increase evident in the grey matter values in the thoracic cord. 

## 4. Discussion

The objective of this study was to characterize MD and FA values across the entire length of the healthy human spinal cord to determine if there are systematic variations in these indices along the cord that must be considered for future DTI studies of spinal cord injury or disease. Our findings demonstrate small but significant differences of the DTI indices between the cervical, thoracic, and lumbar regions of the spinal cord. The MD magnitude ranged from 0.87 to 0.97 (×10^−3^ mm^2^/s) for white matter, and from 1.7 to 1.9 (×10^−3^ mm^2^/s) for grey matter, and these values are consistent with previous measures in the spinal cord, and similar to those reported for similar tissues in the brain [22]. Similarly, measured FA values for white matter and grey matter are consistent with previously reported measures from similar tissues (0.65 to 0.70 for white matter, 0.44 ± 0.08 for grey matter) [17]. Additionally, our results indicate that the FA values from the white matter clusters are significantly greater than the grey matter and CSF clusters, as expected. This finding is consistent with the well-documented anisotropy of apparent water self-diffusion in white matter and further supports the validity of our white matter, grey matter segmentation method. 

Results from this study demonstrate that MD values are consistent along the length of the cord, in all tissue types. These results are supported by the few studies that have compared MD values acquired from different segments of the cord [11, 12]. Unlike MD values, FA values demonstrate variation along the cord, and values from the lumbar, cord differ significantly from values in the cervical and thoracic cord. 

A possible contribution to the slight variation of FA between the cervical/thoracic and lumbar cord is the ratio of white matter to grey matter along the length of the cord [23]. The ratio of grey matter to total transverse area within the cord is higher in the cervical cord (18%) compared to the thoracic cord (13.2%) and is greatest in the lumbar cord (36.3%) [24]. Grey matter has a higher MD and lower FA values compared to white matter; therefore, it is hypothesized that variations in the percentage of grey matter within the different regions of the cord may account for variations in the DTI indices. In support of this hypothesis, a previous study demonstrating variations within FA measures within the cervical cord demonstrated that decreases in FA were consistent with regions of the cervical cord known to have slight increases in grey matter [11]. The FA values we measured from three regions of the cord were observed to be correlated with the percentage of grey matter (linear regression, *R* ^2^ = 0.998, based on three data points). Therefore, changes in the white, grey matter ratio may account for the variation in DTI indices evident in our study. 

Additionally, cord motion caused primarily by the CSF flow resulting from the cardiac cycle has been shown to influence diffusion measures [15]. Previous studies have demonstrated that CSF pulsation varies according to superior-inferior location within the spinal canal and that several diffusion indices (trace, primary, and tertiary eigenvalues) are higher in locations with the greatest cardiac cycle-related motion (C4, C6, T1) [14, 15]. Therefore, diffusion values may be systematically overestimated because of the motion-related errors, and variations in indices along the cord may result from variation in cord motion. We applied cardiac gating in order to limit our acquisition window to the most quiescent period of the cardiac cycle to reduce the amount of cord motion [13, 15]. We, therefore, conclude that the cardiac gating employed in this study sufficiently reduced the motion-related errors and that our indices reflect true anatomical differences in cord diffusion across the regions of the cord. 

By comparing the distribution of MD versus FA between the different regions of the cord, we have obtained accurate measures of DTI indices (as evidenced by the consistency with previous studies) but have avoided manual segmentation of the specific tissues which may be user dependent and can introduce additional variance into the measured MD and FA values. This analysis method may prove useful for patient populations in which a manual segmentation is impracticable, as changes in the distribution of the DTI indices may provide critical clinical information. However, regardless of the segmentation approach, partial volume effects, which arise when the signal from a voxel is composed of signals from multiple tissues, cannot be avoided. Our results revealed a continuous distribution of FA versus MD values, accurately reflecting the DTI indices from voxels that contain mixed tissues. It is important to consider that voxels containing both white matter and CSF would result in FA and MD values that fell into the range of our grey matter cluster. However, visual examination of the spatial location of the centroids from the grey matter cluster indicates that we have accurately detected some grey matter regions although the edge of the cord (CSF/WM boundary) also contributed.

The wide-spread use of spinal cord DTI is currently limited by the absence of standardized methods for data acquisition and analysis as well as by the lack of comprehensive normative data for clinical comparison. Given the sensitivity of FA and MD calculation to different methods of acquisition (ex: employing cardiac gating or not), a more thorough characterization of the influence of using different pulse sequences, different field strengths, and different DTI analysis techniques would be advantageous to explore to better our understanding of how to both acquire and analyze DTI data, but also how to interpret and compare across different results.

 In conclusion, this study characterized diffusion measures along the entire healthy spinal cord and demonstrated slight variations (albeit insignificant when corrected for multiple comparisons) between the three regions of the cord. However, it is possible that this was a consequence of the specific population studied, given that we only had 9 participants. Compared to the large changes in DTI indices that result from injury, FA and MD measures in the healthy cord in the present study were observed to be consistent across regions [8]. Therefore, comparisons between injured and healthy DTI indices are expected to be valid between different sections of the cord.

## Figures and Tables

**Table 1:** MD and FA values for each tissue type along each region of the cord.

	WM cluster		GM cluster		CSF cluster	
	MD	FA	MD	FA	MD	FA
C	0.97 ± 0.32	0.70 ± 0.08^‡^	1.8 ± 0.61	0.45 ± 0.07	3.06 ± 1.02	0.28 ± 0.08
T	0.96 ± 0.32	0.71 ± 0.08^‡^	1.9 ± 0.34	0.44 ± 0.08	3.12 ± 0.61^‡^	0.27 ± 0.07
L	0.87 ± 0.32	0.65 ± 0.08^∗†^	1.7 ± 0.34	0.44 ± 0.08	2.72 ± 0.33^†^	0.30 ± 0.07

Summary of MD (×10^−3^ mm^2^/s) and FA values measured in the cervical (C), thoracic (T), and lumbar (L) spinal cord. The symbols *, ^†^, and ^‡^ indicate significant differences (*P* < 0.001 uncorrected) from values measured in cervical, thoracic, and lumbar regions, respectively. No significant differences were detected at *P* < 0.05 when a Bonferroni correction was applied.
